# The emerging role non-coding RNAs in B cell-related disorders

**DOI:** 10.1186/s12935-022-02521-1

**Published:** 2022-02-22

**Authors:** Soudeh Ghafouri-Fard, Tayyebeh Khoshbakht, Bashdar Mahmud Hussen, Mohammad Taheri, Elena Jamali

**Affiliations:** 1 Department of Medical Genetics, School of Medicine, Shahid Beheshti University of Medical Sciences, Tehran, Iran; 2 Men’s Health and Reproductive Health Research Center, Shahid Beheshti University of Medical Sciences, Tehran, Iran; 3 Department of Pharmacognosy, College of Pharmacy, Hawler Medical University, Kurdistan Region, Erbil, Iraq; 4 Center of Research and Strategic Studies, Lebanese French University, Erbil, Kurdistan Region Iraq; 5 Skull Base Research Center, Loghman Hakim Hospital, Shahid Beheshti University of Medical Sciences, Tehran, Iran; 6 Institute of Human Genetics, Jena University Hospital, Jena, Germany; 7 Department of Pathology, Loghman Hakim Hospital, Shahid Beheshti University of Medical Sciences, Tehran, Iran

**Keywords:** B cell, Immune system, lncRNA, miRNA, Expression

## Abstract

Long non-coding RNAs and microRNAs have recently attained much attention regarding their role in the development of B cell lineage as well as participation in the lymphomagenesis. These transcripts have a highly cell type specific signature which endows them the potential to be used as biomarkers for clinical situations. Aberrant expression of several non-coding RNAs has been linked with B cell malignancies and immune related disorders such as rheumatoid arthritis, systemic lupus erythematous, asthma and graft-versus-host disease. Moreover, these transcripts can alter response of immune system to infectious conditions. miR-7, miR-16-1, miR-15a, miR-150, miR-146a, miR-155, miR-212 and miR-132 are among microRNAs whose role in the development of B cell-associated disorders has been investigated. Similarly, SNHG14, MALAT1, CRNDE, AL133346.1, NEAT1, SMAD5-AS1, OR3A4 and some other long non-coding RNAs participate in this process. In the current review, we describe the role of non-coding RNAs in B cell malignancies.

## Introduction

B cells are a subset of immune cells which contribute in the induction of humoral responses. These cells can be sub-classified to three classes based on their ontogeny and anatomic localization. B1 cells are produced from B1 progenitors. B cell progenitor cells of the bone marrow can produce the marginal zone and follicular B cells. Notably, B1 lymphocytes are originated from B1 progenitor cells which reside in the hepatic tissue during the fetal period. These cells preserve their self-renewal capacity after the neonatal time. B2 cells are developed from transitional 2 B cells originating from bone marrow precursors and have sustained output all through the adulthood period [1]. Abnormal development of B cells can result in human disorders including immune deficiency, autoimmunity, or allergy [2].

B cells are the principal source of antibodies. A typical example of antibodies produced by B1 lymphocytes is the naturally produced antibodies against ABO blood groups [3]. B1 cells can produce IgM antibodies contributing in the maintenance of tissue homeostasis due to their aptitude to bind with reformed self-antigens. These antigens include those produced in the process of cell apoptosis, ischemic damage and oxidative insult in atherosclerosis [7]. Besides, polyreactive IgA antibodies produced by B1 and follicular B cells participate in the mucosal immunity [4].

In addition, B cells also have an immunomodulatory effect through regulation of immune responses via producing cytokines that impede initiation or progression of immune-related disorders [1].

Several non-coding RNAs have been demonstrated to be involved in the regulation of function of different classes of B cells, thus contributing in the pathoetiology of related diseases. In fact, three classes of non-coding RNAs, namely long non-coding RNAs (lncRNAs), microRNAs (miRNAs) and circular RNAs (circRNAs) have been vastly investigated in the context of B cell-related disorders. LncRNAs have sizes more than 200 nucleotides, share many features with mRNAs and regulate gene expression at different levels [5]. CircRNAs are a group of transcripts that are produced through 3’-5’ ligation of a single RNA molecule. These transcripts have also regulatory functions on gene expression. They can also produce polypeptides [6]. Finally, miRNAs are transcripts with sizes about 22 nucleotides that suppress expression of mRNAs or degrade them through a base-pairing mechanism [7].

Through RNA sequencing and de novo transcript assembly methods, Brazão et al. have recognized more than 4500 lncRNAs which are expressed in different phases of development and activation of B cells [8]. Notably, the majority of these transcripts have not been formerly identified, even in the process of commitment of T cells. About one-fifth of these lncRNAs have been found to be either enhancer- or promoter-associated transcripts. Moreover, the B-cell lineage activating transcription factor PAX5 has been shown to directly regulate expression of tens of lncRNAs in pro-B and mature B cells as well as in acute lymphoblastic leukemia (ALL) [8].

In the current paper, we discuss the effects of non-coding portion of the genome on function of this class of immune cells in different contexts. We also explain the impact of dysregulation of non-coding RNAs in the development of B cell-related disorders, particularly malignant conditions as well as imbalances of immune responses. Identification of the role of these transcripts in these conditions would help in design of targeted therapies for these disorders.

## Contribution of miRNAs in the regulation of B cell functions and related disorders

Several miRNAs have been found to affect function of B cells. This process has been mostly evaluated in the context of immune-related disorders and cancers. For instance, miR-7 has been shown to influence expression of PTEN in B cells. Expression of this miRNA has been increased in MRL^lpr/lpr^ mouse model of lupus. Treatment with miR-7 antagomir has decreased disease manifestations in these animals. miR-7-related inhibition of PTEN/AKT signaling has enhanced differentiation of B cells into plasmablasts/plasma cells. Moreover, miR-7 silencing has reduced spontaneous formation of germinal center and normalized B cell subtype fractions in the spleen. In addition, miR-7 antagomir has decreased phosphorylation of STAT3 and IL-21 synthesis. Taken together, miR-7 has an important role in regulation of PTEN expression and functions of B cells [9].

Tan et al. have assessed miRNA profiles of naïve, germinal center and memory B cells. They have reported elevation of numerous miRNAs in germinal center B cells. miR-17-5p, miR-106a and miR-181b have been among mostly up-regulated miRNAs in these cells. miR-150 has been a miRNA with high expression in all three B-cell subsets. However, its expression has been found to be lower in germinal center B cells compared with naïve and memory B cells. Notably, expressions of miR-17-5p, miR-106a and miR-181b have been gradually decreased from the dark to the light zone of germinal center. Expression of miR-150 has been inversely correlated with c-Myb and Survivin levels in tonsil tissues, implying potential inhibition of these genes by miR-150 [10].

Several other miRNAs have been found to affect pathogenesis of diffuse large B-cell lymphoma (DLBC). A number of miRNAs have been shown to be dysregulated in these patients. For instance, expression of miR-16–1 has been found to be significantly lower in DLBC patients compared to controls in a single study [11]. Another study has shown differential expression of miR-197 in DLBCL versus controls. While expression levels of miR-197 have not been correlated with clinicopathologic parameters such as international prognostic index, down-regulation of this miRNA has been associated with disease progression in patients treated with rituximab plus cyclophosphamide, doxorubicin, vincristine, and prednisone. Down-regulation of miR-197 levels could predict shorter progression-free survival in this subgroup of patients as well as non-germinal center B-like subgroup. Cell line studies have shown that miR-197 can enhance doxorubicin-associated apoptosis in SUDHL9 cells but not in OCI-Ly1 cells [12].

Another study in the context of sepsis has shown up-regulation of miR-19a in B cells. Moreover, in vitro studies have confirmed over-expression of this miRNA in activated B cells. Expression of CD22 has been initially increased but afterwards reduced. Notably, up-regulation of miR-19a has led to activation of BCR signaling, whereas up-regulation of CD22 has resulted in the attenuation of the effects of miR-19a and enhanced its expression. Taken together, miR-19a and CD22 contribute in establishment of a feedback circuit for B cell responses in sepsis, which can be considered as a putative target for re-establishment of immune homeostasis [13].

miR-30a is another miRNA that participates in the activation of B cells. This miRNA can specifically bind the 3'-UTR of Lyn transcript to inhibit its expression. miR-30a expression has been found to be elevated in B cells of patients with systemic lupus erythematous (SLE) compared with controls. Moreover, its levels have been negatively correlated with Lyn levels in B cells. Up-regulation of miR-30a has promoted proliferation of B cells and release of IgG antibodies. Thus, up-regulation of miR-30a can reduce Lyn levels in B cells, indicating its role in induction of B cell hyperactivity in SLE [14].

miR-155 is an example of miRNAs whose functions have been evaluated in different contexts such as rheumatoid arthritis [15], DLBC and non-Hodgkin lymphoma [16] as well as chronic psychological stress [17]. In B cell malignancies, higher levels of miR-155 have been correlated with the presence of B symptoms, involvement of extranodal sites, and high ECOG score [16].

**Fig. 1:**
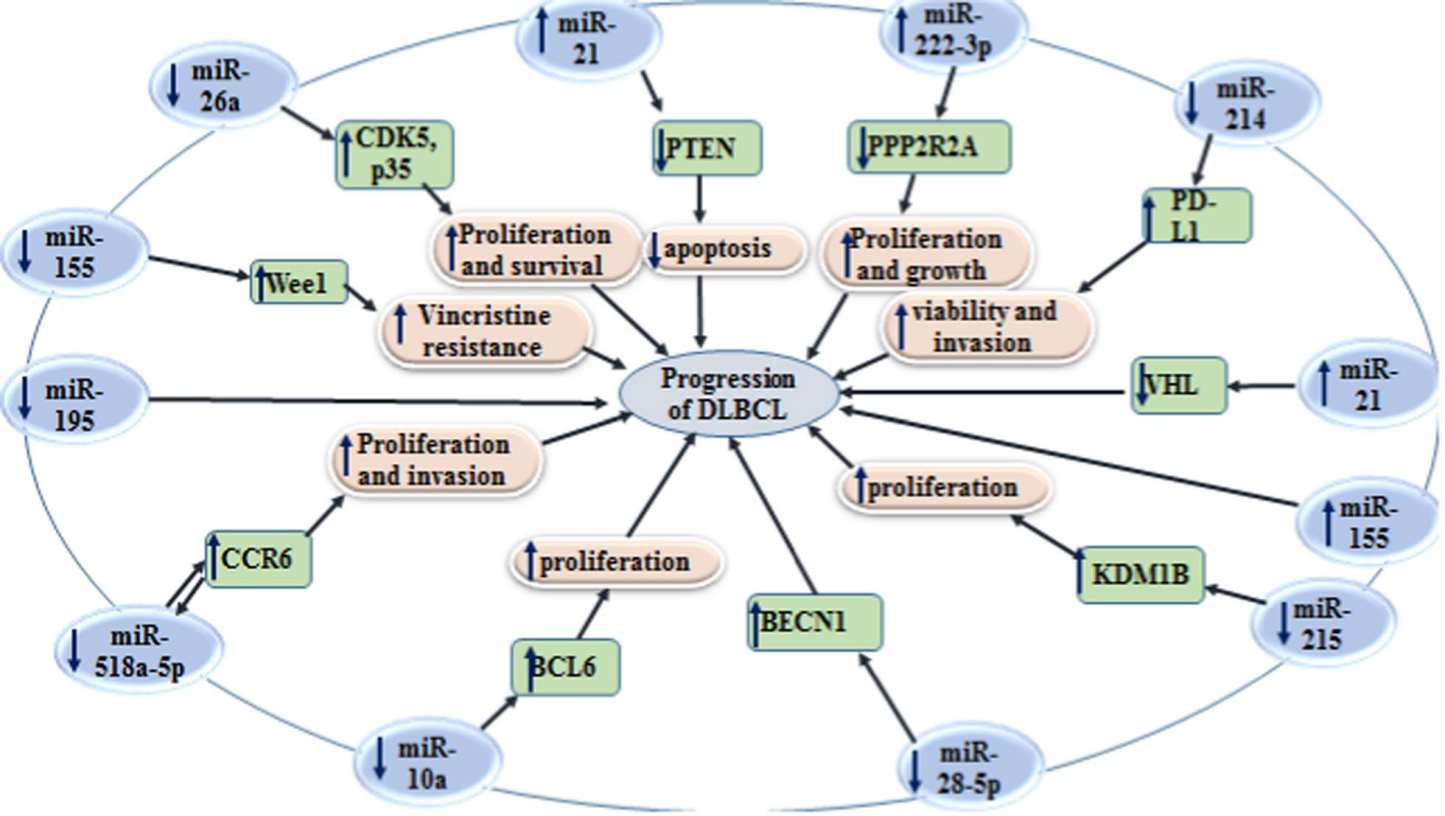
The impacts of miRNAs on regulation of their target genes in the context of DLBCL. Detailed information about these miRNAs is presented in Table 1.

Figure 1 depicts the impacts of miRNAs on regulation of their target genes in the context of DLBCL.

**Table 1:** miRNAs and B cell functions

microRNA	Expression pattern	Disease/	Sample	Cell line	Interaction	Signaling pathway	Function	References
*Human studies*								
miR-16–1	↓	DLBC	40 untreated patients diagnosed with DLBC and 15 healthy controls	CD19( +) and CD20( +) cells	–	–	–	[11]
miR-15a	no difference	DLBC	40 untreated patients diagnosed with DLBC and 15 healthy controls	CD19( +) and CD20( +) cells	–	–	–	
miR-150	↑ (lower in GC B cell than the other two subsets)	chronic tonsillitis	children with chronic tonsillitis	Naïve B cells, GC B cells and memory B cells	c-Myb, Survivin and Foxp1	–	↑ miR-150: ↑ the amount of apoptotic/death cells, ↓ c-Myb, Survivin and Foxp1	[10]
miR-155	↑ (abundantly in synovial B cells)	RA	27 patients with ERA and 33 patients with LSRA, 14 patients with osteoarthritis, 9 healthy controls	B cells, CD19 + cells, synovial B cells	PU.1	–	↑ RA B-cell activation associated with autoantibody production ∆ miR-155: ↓ antibody synthesis	[15]
viral miR-BHRF1	↓	EBV-immortalized B lymphoblastic cell malignancy	–	Ramos and BJAB, Manassas, VA, B95.8, HEK293	SMAD3, JUN, and COL1A	TGF-β signaling pathway	LA: ↓ viral miR-BHRF1-1: ↑ adhesion and the growth of EBV-infected B cells	[18]
miR-28	↓	BL	–	GC B cells, HEK293T cells and B-cell lines	MAD2L1, BAG1, MYC	–	↓ proliferation and clonogenic properties of BL cells, MYC-induced transformation	[19]
miR-19a	↑	sepsis	64 patients with SIRS and 15 healthy controls	PBMCs	CD22	BCR signaling	↑ BCR signaling	[13]
miR-30a	↑	SLE	patients with SLE and healthy controls	Daudi and Raji B cell lines	Lyn	–	↑ B cell proliferation and the production of IgG antibodies through inhibiting Lyn	[14]
miR-194	↓	PTLD	PBMC or lymph node from six PTLD patients and 4 healthy blood donors	AB5, JB7, JC62, MF4, VB5, ZD3 derived from PBMC or lymph node of six PTLD patients and B lymphoblastoid cell lines isolated from 4 healthy blood donors	IL-10	–	Expression of microRNA-194 was suppressed by EBV microRNA-194 inhibited IL-10 expression, so reduced proliferation and promoted apoptosis of EBV( +) B cell lymphoma lines	[20]
miR-125b	↑	–	–	murine Bcl1.3B3 B lymphoma and the human U266 multiple myeloma cell lines	BLIMP-1 and IRF-4	–	↓ differentiation of GC centroblasts and myeloma cell survival through inhibiting BLIMP-1 and IRF-4 translation	[21]
miR-148b	↓	BCL	Peripheral blood from 21 patients with BCL and 18 healthy controls, Lymphatic tissue from 30 patients with BCL and 20 healthy controls, male BALB/c nude mice	Raji and SU-DHL-10 human BCL cell lines, HEK-293 T	Bcl-w	–	↓ cell viability, colony formation, and ↑ apoptosis in irradiated BCL cells, ↓ growth of tumors in nude mice (↑ radiosensitivity of BCL cells)	[22]
miR-197	↓	DLBCL	51 patients with DLBCL	SUDHL9 and OCI-LY1 human DLBCL cell lines	–	–	↑ miR-197: ↑ effects of doxorubicin on reducing cell viability and enhancing apoptosis	[12]
miR-124	↓	DLBCL	–	OCI-Ly1 and HBL1	p65	TAK1/IKKα-IKKβ/IκBα and MAPK/p65 signaling pathways, NF-κB signals	↓ cell proliferation and survival	[23]
miR-17–92	↑	B-NHL	71 patients with B-NHL, 5 patients with reactive hyperplasia lymph nodes as controls, female Balb/c nude mice	WT, KO and TG lymphoma cells and reactive hyperplasia lymph cells obtained from mice	–	–	↑ miR-18: ↓ OS ↑ miR-19 and miR-92a: ↓ OS and EFS ↑ miR-17–92: ↓ the duration of incubation required for visualization of the xenograft tumor	[24]
miR-155	–	DLBCL	76 patients with DLBCL	HEK293T, RIVA, U2932, DHL4, HBL-1, Ly7, Ly18, and Ly19 cell lines	DEPTOR and c-CBL	BCR signaling	∆ mir-155: ↓ expression of NFkB target genes and ↑ sensitivity DLBCL cells to ibrutinib Low expression of DEPTOR (a target of mir-155) increased the migration of DLBCL cells toward the CXCL12 gradient and modulated cytokine production	[25]
miR-320d	↓	DLBCL	85 patients with DLBCL, 19 samples with lymph node reactive hyperplasia as controls	OCI-LY1 (GCB subtype) and NU-DUL-1 (ABC subtype) human DLBCL cell lines	CDK6	–	↓ proliferation in GCB type of DLBCL cells and ↓ CDK6 expression	[26]
miR-195	↓	DLBCL	60 patients with DLBCL and 30 healthy controls		–	–	Expression levels of miR-195 closely correlated with tumor diameter, IPI score and Ann Arbor stage Patients with high levels of miR-195 had longer OS	[27]
miR-155	↓ in vincristine- resistant DLBCL cell lines	DLBCL	73 patients with DLBCL, GEO database: data (GSE10846 and GSE31312)	U-DHL-5 and OCI-Ly7 GCB-DLBCL cell lines, RIVA and NU-DHL-1 ABC cell lines	Wee1 (a direct target of miR-155)	–	↑ sensitivity to vincristine Expression level of miR-155 was strongly correlated with superior survival for R-CHOP-treated patients of the GCB subclass	[28]
miR-153-3p	↓ in IM-resistant CML cells	CML	Blood samples obtained from 44 CML patients	human KBM5, K562 and IM-resistant KBM5R, K562R CML cell lines	Bcl-2 (a direct target of miR-153-3p)	–	↑ miR-153-3p: ↑ IM sensitivity and ↓ the survival rate of IM-resistant CML cells ↓ autophagy caused by IM in IM-resistant CML cells	[29]
miR-30c	↑ in patients with SCNSL	PCNSL, SCNSL	61 CSF samples from patients with PCNSL and 14 samples from SCNSL	–	–	–	miR-30c could act as a biomarker to distinct PCNSL from SCNSL	[30]
miR-155	↑	NHL and DLBCL	84 patients with B-cell NHL and 15 healthy controls	–	–	–	Higher levels of miR-155 were correlated with the presence of B symptoms, involvement of extranodal sites, and high ECOG score In DLBCL, higher levels of miR-155 were correlated with non-germinal B-cell-like type, the presence of B symptoms, involvement of extranodal sites, and higher IPI and ECOG scores ↑ miR-155; ↑ lower event-free survival	[16]
hsa-miR-34a-5p	↑	DLBCL	six serum samples from patients with DLBCL and 3 healthy control	–	TP53	p53 signaling pathway	hsa-miR-34a-5p was involved in 15 pathways such as the p53 signaling pathway	[31]
hsa-miR-323b-3p	↓	DLBCL	six serum samples from patients with DLBCL and 3 healthy control	–	–	–	hsa-miR-323b-3p was involved in four pathways such as pathways in cancer	
hsa-miR-431-5p	↓	DLBCL	six serum samples from patients with DLBCL and 3 healthy control	–	FYN	–	regulating FYN	
miR-155	↑ in EBV-infected B cells	lymphoma	–	DG75 cell line originated from an EBV-negative BL, DG75 RBPJ knockout cell line derived from DG75 wt parental cells, IB4 and GM12878 obtained from Coriell Cell Repositories (EBV-immortalized lymphoblastoid cell lines)	EBNA2, IRF4, RBPJ	–	↑ the growth of EBV-infected B cells	[32]
miR-3173	↓	B-ALL	GEO database (GSE4732, GSE4475, GSM565540) 135 children with B-ALL and 97 healthy controls plus 430 children with B-ALL and 340 healthy controls	CCRF-SB and SUP-B15 human B-ALL cell lines	PTK2 (a direct target of miR-3173)	–	↓ proliferation, migration and invasion	[33]
miR-21	↑	B-ALL	75 children with B-ALL and 50 healthy controls	–	–	–	Lower DFS and OS	[34]
miR-21	↑	DLBCL	36 tissue samples from 26 patients with DLBCL and 10 healthy controls	CRL-2630	PTEN	–	higher in stage III/IV patients, ↓ apoptosis (by regulating the expression of PTEN)	[35]
miR-222-3p	↑	DLBCL	74 patients with initial diagnosis of ABC-type DLBCL, 26 patients with pathological diagnosis of reactive lymphoid hyperplasia as controls, SPF BALB/c nude mice	HMy2.CIR human normal B-cell immortalized cell line, DLBCL cell line, germinal central B-cell (GCB)-like OCI-Ly19 and SU-DHL-4, and ABC-like OCI-LY10 and U2932	Phosphatase 2 regulatory subunit B alpha (a direct target of miR-222-3p)	–	↑ proliferation, invasion and tumor growth, ↓ apoptosis	[36]
miR-29a	↓	SLE	peripheral blood of 66 patients with SLE and 10 healthy controls	Raji,	CRKL (a target gene of miR-29a)	–	↓ the production of IgG (by regulating CRKL)	[37]
hsa-miR-223-3p and hsa-miR-21-5p	↓ from stage I to stage III of PBC	PBC	Peripheral B cells from 72 PBC patients and 15 healthy controls	–	mutual 4 target genes: TGFBR2, MEF2C, FOXP1 and RBPJ	–	modulating B cell functions, such as B-cell signal transduction, differentiation, migration, and apoptosis in GO categories	[38]
miR33b, miR96, and miR503	↓	Lymphoma	–	JeKo-1, Pfeiffer, SUDHL-2, PDX, and A20	PRMT5, CYCLIN D1 and c-MYC (target genes of miR33b, miR96 and miR503)	–	↓ lymphoma cell survival	[39]
miR-214	↓	DLBCL	15 pairs of DLBCL tissues and ANCTs, female BALB/c nude mice	OCI-Ly3, SU-DHL-2 and OCI-Ly10 human DLBCL cell lines, a normal B-cell line (NBC) and HEK-293 T	PD-L1	–	↓ viability and invasion, ↑ apoptosis	[40]
miR-107	↓	ABMR	19 patients with ABMR and 20 healthy controls	B lymphocytes, Daudi, Raji, and HEK-293	ATG12	–	↑ miR-107: ↓ formation of autolysosomes in B lymphocytes of recipients, autophagy, and secretion of IgG and IgM antibodies	[41]
miR-92a	↑ in PMBL than in DLBCL, but not in cHL	PMBL, DLBCL, cHL	40 patients with PMBL, 20 patients with DLBCL, and 20 patients had with cHL	Karpas-1106P, SU-DHL-5	FOXP1 (a target of miR-92a)	–	↓ proliferation, ↑ apoptosis,	[42]
miR-21	↑	DLBCL	45 samples of lymphoma tissues from patients with DLBCL	SU-DHL-8, OCI-LY1, and SU-DHL-10	VHL (a target of miR-21)	–	Curcumin decreased the proliferation, migration, and invasion abilities and increased apoptosis by suppressing miR-21	[43]
miR-155	↑	DLBCL	76 patients with DLBCL and 40 samples with	DB cells	–	–	↑ migration and invasion, ↓ apoptosis	[44]
miR-215	↓	DLBCL	50 patients with DLBCL and 30 samples with RPL	SU-DHL-4 cells	KDM1B	–	↓ proliferation and ↑ apoptosis Low levels of miR-215 were correlated with shorter 5-year OS	[45]
miR-155	↑ in tonsillar memory B cells and PBMCs activated with CpG	DS	–	PBMCs and Tonsils from healthy controls and children with DS	AID (a target of miR-155)	–	miR-155 played a role in DS-associated dementia and leukemia	[46]
miR-125b	↑ in tonsillar memory B cells and plasma cells	DS	–	PBMCs and Tonsils from healthy controls and children with DS	–	–	miR-125b played a role in DS-associated dementia and leukemia	
miR-98	↑	asthma	20 patients with asthma and 20 healthy controls	PBMCs from healthy controls and patients with asthma	TSP1	–	IL-13 decreased TSP1 expression through up-regulating expression of miR-98 in B cells	[47]
miR-28-5p	↓	DLBCL	–	OCI-LY7 human GCB-type DLBCL cell line and HEK-293 T	BECN1 (a direct target of miR-28-5p)	–	Curcumin: ↑ miR-28-5p: ↓ proliferation and autophagy, ↑ apoptosis	[48]
miR-21	↑	DLBCL	53 patients with DLBCL	–	Ki-67	–	High expression levels of miR-21 was correlated with poor response to treatment	[49]
miR-10a	↓	DLBCLs	9 patients with DLBCL and 9 samples with RLH as controls	OCI-LY7 and OCI-LY3 human DLBCL cell lines and HEK293T	BCL6 (a direct target of miR-10a)	–	↓ proliferation, ↑ apoptosis	[50]
miR-125a	↓	AML	–	HL60	p53, Bcl-2, c-myc	NF-κ Pathway	↑ miR-125a: ↓ viability and invasion, ↑ apoptosis	[51]
let-7b-5p	↑	ITP	61 patients with ITP and 31 healthy controls	PBMC from samples, peripheral CD19 + cells	BAFF, BAFF-R, NF-κB2 p100, Bcl-xL	–	↑ B cell survival, ↑ BAFF-R and BAFF levels, ↑ phosphorylation of NF-κB2 p100	[52]
miR-27a	↑	KD	23 children with acute KD and 23 healthy controls	PBMCs from samples, PurifiedCD19 + B cells, CD14 + monocyte cells	IL-10	–	↑ monocyte-mediated TNF-α release, ↑ monocyte-mediated inflammatory responses via inhibiting the regulatory function of B10 cells	[53]
miR-17–92	–	–	C57BL/6 mice	38c13 cells, HEK, CD19KO B cells	c-Myc, PTEN (a target of miR-17–92)	PI3K/Akt/Foxo1 pathway	∆ miR-17–92: ↑ RAGs expression (post-translationally through Foxo1) miR-17–92: ↓ B cell development	[54]
miR-4638-5p	↓ in ERG + DLBCL	DLBCL	126 patients with DLBCL (in Kaplain‐Meier survival analysis) and 94 patients with DLBCL (in the clinicopathologic correlation study)	–	ERG	–	More mutations in genes important in cell cycle control, B-cell receptor-mediated signaling and degradation of β-catenin were seen in ERG + DLBCL more likely harbors	[55]
miR-518a-5p	↓	DLBCL	56 samples with DLBCL and 29 samples with RLH as controls	HMy2.CIR normal B cell line, SU-DHL-2 and SU-DHL-6 DLBCL cell lines	CCR6, (a direct target of miR-518a-5p)	JAK2-STAT6 signalling pathway	There is a negative regulatory feedback loop between miR-518a-5p and CCR6 in DLBCL ↑ miR-518a-5p: ↓ proliferation and invasion, ↑ apoptosis	[56]
miR-296-5p	↑	DLBCL	–	DLBCL-DB cells	–	–	∆ miRNA-296-5p: ↓ proliferation and migration, apoptosis did not change	[57]
miR-34a	↓	DLBCL	65 patients with DLBCL and 22 samples with LRH as controls	–	↑ BCL-2	–	Patients with high levels of miR-34a had longer OS	[58]
miR-224	↓	DLBCL	76 patients with DLBCL and 41 healthy controls	–	PIK3CD (a direct target of miR-224)	–	↑ miR-224: ↓ proliferation and invasion, ↑ apoptosis	[59]
miR-451a	↓	DLBCL	89 patients with DLBCL and 48 healthy controls	–	–	–	The efficacy of rituximab combined with chemotherapy can be evaluated by miR-451a as an indicator	[60]
miR-152-3p	↑	SLE	30 female patients with active SLE and 30 female healthy controls	SLE B-cells	KLF5 (a direct target of miR-451a), BAFF	–	∆ miR-152-3p: ↓ self-reactivity of SLE B-cells, and ↓ autoantibody production	[61]
miR-28	↓ in GC-derived neoplasms	Non-Hodgkin lymphoma	human primary GC-derived B-cell neoplasms (GSE 29,493), NSG mice	naïve B cells (CD19 + GL7 −), GC B cells (CD19 + GL7 +), and post-GC B cells (CD19 + GL7 − IgA +) from Peyer’s patches Ramos and Raji BL GC-derived B-cell lines and MD901 DLBCL cell line	–	BCR signaling	Downregulation of miR-28 expression is correlated with GC B-cell transformation ↑ miR-28: ↓ proliferation and survival	[62]
miR-98	↑	heart transplantation	peripheral blood samples from 20 patients with advanced heart failure before and after and 20 healthy controls, male BALB/c mice and male C57/B6 mice	peripheral blood mononuclear cells were isolated from the blood samples	↓ IL-10	–	The levels of miR-98 and serum levels of cortisol were increased in peripheral B cells after heart transplantation Cortisol-suppressed IL-10 expression was mediated by miR-98	[63]
miR-21-5p	↑ in cHL than GC-B cells	cHL	–	L540, KM-H2, L1236, L428 and L591, SUPHD1 CHL cell lines and HEK-293 T	PELI1	–	∆ miR-21-5p: ↓ growth, ↑ apoptosis	[64]
miR-29a	↓	Arthritis	–	miR-29a knockout mice	–	–	↑ B-cell activation and germinal center production	[65]
miR-126	↓ in MLL-AF4 ALL	ALL	Congenic mice	Ebf1 − / − hematopoietic progenitor (Lin −) cells were isolated from the Ebf1 − / − livers of 14 d postcoitum embryos	IRS-1	–	miR-126 drived B-cell myeloid biphenotypic leukemia differentiation toward B cells. (↑B cells) miR-126 could partly rescue failed B-cell lineage development and specification	[66]
miR-212	↑	Autoimmune disease and cancer	C57BL/6 WT and miR-212/132 − / − mice	HEK293T, primary splenic B cells	–	BCR signaling	BCR activation: ↑ miR-212	[67]
miR-132	↑	Autoimmune disease and cancer	C57BL/6 WT and miR-212/132 − / − mice	HEK293T, primary splenic B cells	Sox4	BCR signaling	BCR activation: ↑ miR-132 ↓ early B cell development, ↑ apoptosis in primary bone marrow B cells ∆ miR-132: B cell recovery after antibody-mediated B cell depletion ↓ B cell leukemia development	
mir-23a cluster	–	–	mirn23a − / − mice and WT C57BL/6 mice	A20 and EML, 32Dcl3	Ebf1, Pax5, Mef2c, Ikzf1, FoxO1, Trib3	–	∆ mirn23a: ↑ B cells, ↑ B lymphopoiesis, ↑ T1 population of transitional B cells, ↑ CLP population and ↓ myeloid cells, ↓ myelopoiesis, ↓ myeloid progenitor populations B cells with mirn23a − / − genotype secrete normal levels of IgG, proliferate normally, and could differentiate into short-lived effector plasma cells in response to antigen	[68]
miR-148a	**↑**	Lupus	gMb-macroself, Gadd45a − / − , Bcl2l11 − / − , Ptenfl/fl, Cd19-Cre, Tnfrsf1b − / − mice, and CD45.1 + C57BL/6 J mice	HEK293T, splenic B cells (CD19 +) and BM B cell precursors (CD19 + IgM −) from CD45.1 + C57BL/6 J mice	Gadd45α, PTEN, Bim	–	miR-148a was found to be a regulator of B cell tolerance by promoting the survival of immature B cells and accelerating the development of autoimmunity by suppressing the expression of Gadd45α, PTEN, Bim	[69]
miR-17–92	**↑**	cGVHD	miR-17–92 conditional knockout mice (BALB/c mice)	donor BM-derived cells (Ly5.1 +) in peripheral blood and spleen, miR-17–92–deficient B cells,	–	–	miR-17–92 increases the pathogenicity of B cells, promoted GC responses and B-cell function, the development of BO and reduced proteinuria/ascites	[70]
miR-125b	Epigenetic silencing of miR-125b is necessary for normal B-cell development	–	WT and Eμ/miR-125b-Tg mice	HEK293T, bone marrow sinusoidal and parenchymal B cells from Eμ/miR-125b-Tg mice and littermate controls	S1PR1, IRF4	–	Expression of miR-125b impaired B-cell egress from the bone marrow to peripheral blood	[71]
miR-26a	↓ in DLBCL cell lines compared to B lymphocytes	DLBCL	NOD/SCID mice	SU-DHL-4, SU-DHL-6, SU-DHL-16 GCB cell lines and SU-DHL-2, SU-DHL-8, and RCK-8 ABC cell lines	CDK5, p35 (a direct target of miR-26a)	–	↓ DLBCL tumor growth, proliferation, cell-cycle progression, and survival	[72]
miR-155	**–**	–	CD45.1 + congenic mice, SWHEL mice and miR-155–deficient mice (all with the C57BL/6 background)	SWHEL Mir155 + / + or SWHEL Mir155 − / − donor B cells	–	–	miR-155 regulated the early expansion of B-blasts and later on the survival and proliferation of plasmablasts in a B-cell-intrinsic manner miR-155 is required for the optimal proliferation of plasmablast B cells	[73]
miR-181b	↑ in neonatal B cells	–	miR-181a/b1−/− mice; ko mice and miR-181a/b-1 ± mice with C57BL/6 J background	Neonatal and adult B cells	–	–	∆ miR-181b: ↑ class-switch recombination	[74]
miR-155	↓	chronic psychological stress	male C57BL/6 mice	In-vitro-induced GC B cells, Naive B cells, Su-DHL4 cells	FBXO11 (a direct target of miR-155), BCL6	–	Corticosterone treatment: ↓ miR-155: ↓ GC B cell generation and isotope class switching ↑ miR-155: ↓ stress-induced impairment of GC response	[17]
miR-221	–	–	C57BL/6, RAG1−/−(CD45.2, CD45.1) mice	preBI cell lines	PTEN (a target of miR221), CXCL12, Bcl2	PI3K signaling	↑ precursor B-cell retention in the bone marrow, ↑ CXCR4-PI3K mediated Bcl2 upregulation, ↑ early B-cell adhesion capability via PI3K signaling	[75]
miR-92a	↓	DM	Adult mice	Min-6 mouse pancreatic bcells	KLF2 (a direct target of miR-92a)	–	↑ insulin secretion and proliferation, ↓ apoptosis	[76]
miR-15a/16–1	↓	Plasma cell and mature B-cell neoplasms	AIDCre/ + (wild-type [WT]) control and AIDCre/ + ;miR-15a/16-1 fl/fl (knockout [KO]) compound mice with C57BL/6 background	GC B cells from WT and KO mice	–	–	Deletion of the miR-15a/16–1 increased the number of GC B cells, percentage of dark zone B cells, and maturation into plasma cells	[77]
miR-146a	–	–	CD21-cre, Cγ1-cre, CD4-cre, hCD2-cre, and CD40-deficient mice, B-KO/CD40 + / − mice	Naive B cells from unimmunized B-KO mice or WT littermates and GC B cells from corresponding D14 SRBC-immunized mice	–	CD40 signaling pathway	The loss of miR-146a in B cells leaded to the development of spontaneous autoimmunity miR-146a is crucial to maintain optimal B cell responses	[78]
miR-146a	↓	B-cell oncogenesis	Eμ-Myc miR-146a − / − mice	70Z/3 and WEHI-231	Egr1, Blimp1 and Bcl6	–	↓ miR-146a: ↓ survival, ↑ in peripheral blood CD11b + myeloid cells, ↑ mature B-cell phenotype ↑ miR-146a: ↓ cell growth	[79]
miR-21	↑	Lymphoma	NOD-SCID mice	OCI-LY3 and Ramos, OCI-LY10, U2932, Raji, Rec-1, Jeko-1, Maver-1 and JM1, HEK293T	Nl101, Mxd1 (a target of miR-21), c-Myc	–	NL101: ↑ miR-21, c-Myc: ↓ miR-21, miR-21: ↑ proliferation and survival, ↓ apoptosis	[80]
miR-146a	↓	Immune complex glomerulonephritis	miR-146a − / − mice with C57BL/6 background	B lymphocytes were the spleen, HK-2	Kim1/Tim1	–	∆ miR-146a: ↑ numbers of memory B cells and plasmablasts, ↑ glomerular hypercellularity with age , ↓ Bregs and ↓ Kim1/Tim1	[81]
miR-146a	↑	–	Murine OVA-Induced asthma mice, WT and miR-146a TG mice	purified splenic B cells	Smad4 (a direct target of miR-26a), 14–3-3σ	–	↑ class switch and secretion of IgE in B cells	[82]
miR-142	↑	Lymphoma	BMT and transgenic (Eμ/mir142) mice	KHM10B, Raji, KMS12, OCI-Ly8, Hut 78, and Cos7	–	–	In splenic B cells, high expression of Mir142 modified LPS-induced phenotypical changes	[83]
miR-7	↑	SLE	Female MRLlpr/lpr lupus mice	Purified splenic B cells obtained from mice	PTEN	PTEN/AKT signaling	∆ miR-7: ↓ nephritis, ↓ lupus manifestations, ↓ immune Abnormalities, ↓ tfh-derived IL-21 expression, ↓ Abnormal B cell differentiation, normalizes splenic B cell subtypes	[9]
miR-98	↑	Myocarditis	BALB/c mice immunized with MyHC-α	B cells isolated from the mouse hearts with myocarditis	↓ IL-10 (a target of miR-98), TNF-α	–	∆ miR-98: ↓ myocarditis miR-98 is upregulated by TNF-α in B cells	[84]
Let-7	–	–	Lin28a iTg mice, let-7adf cluster KO mice, and let-7bc cluster KO mice	HEK293T	Hk2 (a target gene of Let-7) c-myc (a target gene of Let-7) Slc1a5 and Gls (indirect target genes of Let-7)	–	↓ IgM Production ↓ glycolytic capacity and glucose uptake ↓ glutamine uptake and utilization ↓ B Cell Activation	[85]
Let-7	↑ in thymic B progenitors by in vitro co-culture with IL15, Vitamin-D3, and retinoic acid	–	Foxn1lacZ/lacZ (Z/Z) mice with C57Bl6/J background, Foxn1nude heterozygous (Foxn1 + /nude) mice with C57Bl6/J background, Foxn1lacZ/nude (Z/N) mice, Foxn1 + /lacZ (+ /Z) mice	thymic progenitor B cells	Lin28a, Arid3a	–	↓ B cell production in the thymus, ↓ proliferation of intrathymic progenitor B cells	[86]
miR-191	↑ during B-cell development and differentiation	–	C57BL/6 J and NOD.Cg-PrkdcscidIl2rgtm1Wjl/SzJ (NSG) mice, C57BL/6 mice and miR-191 − / − mice	Primary cells from wild-type or chimeric mice, preB1 cells,	Foxp1, E2A, and Egr1	–	Expression levels of miR-191 are required for efficient B-cell development, V(D)J recombination and IL-7-dependent expansion of preBI cells	[87]
miR-15 family	↓	–	Female C57BL/6 Rag1 − / − mice	wk3, 1587, and 1677 pre‐B cell lines from total bone marrow of SLP‐65 − / − and SLP‐65 − / − LAT − / − mice, respectively, and 1676 and 74 pre‐B cell lines	↑ cyclin E1 and D3	–	The lack of miR-15 family in pre-B cells caused prolonged proliferation, so failed to trigger the transcriptional reprogramming to accompany their differentiation	[88]
mirn23a cluster	↓	–	Wildtype and mirn23a−/− C57BL/6 mice, CD45.1 recipient mice, femurs and tibias of mice	70Z/3, A20 and 32Dcl3 cell lines	↑ Ikzf1, Runx1, Satb1, Bach1 and Bach2 that managed the commitment of MPPs to CLPs ↑ FoxO1, Ebf1, and Pax5 that commited the CLP to the B cell lineage in the absence of mirn23a, EBF1	PI3K/Akt and BMP/Smad signaling pathways	Mirn23a regulated some related transcription factors and signaling pathways to modulate adult hematopoiesis Mirn23a was inhibited by EBF1	[89]

TB, tuberculosis; CTRL, control; BL, Diagnosis; M1, month 1; M6, month 6; GC, germinal center; SLE, systemic lupus erythematosus; DLBC, diffuse large B-cell lymphoma; RA, rheumatoid arthritis; ERA, early rheumatoid arthritis; LSRA, long standing rheumatoid arthritis; BCR, B cell receptor; CLP, common myeloid progenitor; LA, lactic acid; MLL, myeloid/lymphoid leukemia; ALL, acute lymphoblastic leukemia; BL, Burkitt lymphoma; SLE; systemic lupus erythematosus; PTLD, posttransplant lymphoproliferative disorder; EBV; Epstein-Barr virus; BCL, B-cell lymphoma; cGVHD, Chronic graft-versus-host disease; BO, bronchiolitis obliterans; B NHL, B cell non-Hodgkin’s lymphoma; OS, overall survival; EFS, event free survival; WT, wild-type; KO, knockout; TG, overexpression; IM, Imatinib; CML, Chronic myeloid leukemia; PCNSL, Primary lymphomas of the central nervous system; SCNSL, secondary spread of systemic lymphoma to the CNS; CSF, Cerebrospinal fluid; ECOG, Eastern Cooperative Oncology Group; IPI, International Prognostic Index; B-ALL, B-cell acute lymphoblastic leukaemia; DFS, disease-free survival; PBC, Primary biliary cholangitis; ANCTs, adjacent non-cancerous tissues; BMT, bone marrow transplantation; ABMR, Antibody-mediated renal allograft rejection; PMBL, Primary mediastinal large B-cell lymphoma; LNRH, lymph node reactive hyperplasia; RPL, reactive proliferative lymphadenitis; PBMCs, Human peripheral blood mononuclear cells; DS, Down Syndrome; DM, diabetes mellitus; RLH, reactive lymph node hyperplasia; AML, acute myeloid leukemia; ITP, immune thrombocytopenia; KD, Kawasaki disease; IgAN, immunoglobulin A nephropathy; RLH, reactive lymphoid hyperplasia; LRH, lymphonode reactive hyperplasia; cHL, Classical Hodgkin lymphoma

## Contribution of lncRNAs in the regulation of B cell functions and related disorders

Impacts of lncRNAs on B cell functions have been investigated in malignancies, particularly DLBCL. SNHG14 has been shown to be elevated in DLBCL. Its silencing has decreased proliferation, migration and epithelial to mesenchymal transition (EMT) features in these cells. From a mechanistical point of view, SNHG14 could sponge miR-5590-3p and subsequently enhance expression of ZEB1. Moreover, ZEB1 could activate transcriptional of SNHG14 and PD-L1 to increase immune evasion in these cells. Cumulatively, SNHG14/miR-5590-3p/ZEB1 axis can promote progression of DLBCL and immune evasion in a positive feedback loop. This axis can regulate PD-1/PD-L1 checkpoint [90].

Another study has shown up-regulation of MALAT1, PD-L1 and CD8 in DLBCL tissues, parallel with down-regulation of miR-195. Mechanistically, MALAT1 has been shown to sponge miR-195 to influence PD-L1 levels. MALAT1 silencing has enhanced miR-195 levels and reduced PD-L1 levels. Moreover, MALAT1 silencing has suppressed proliferation, migratory potential and immune escape aptitude of DLBCL cells while increasing their apoptosis. MALAT1 silencing has also inhibited EMT features through modulation of Ras/ERK signaling [91].

**Fig. 2:**
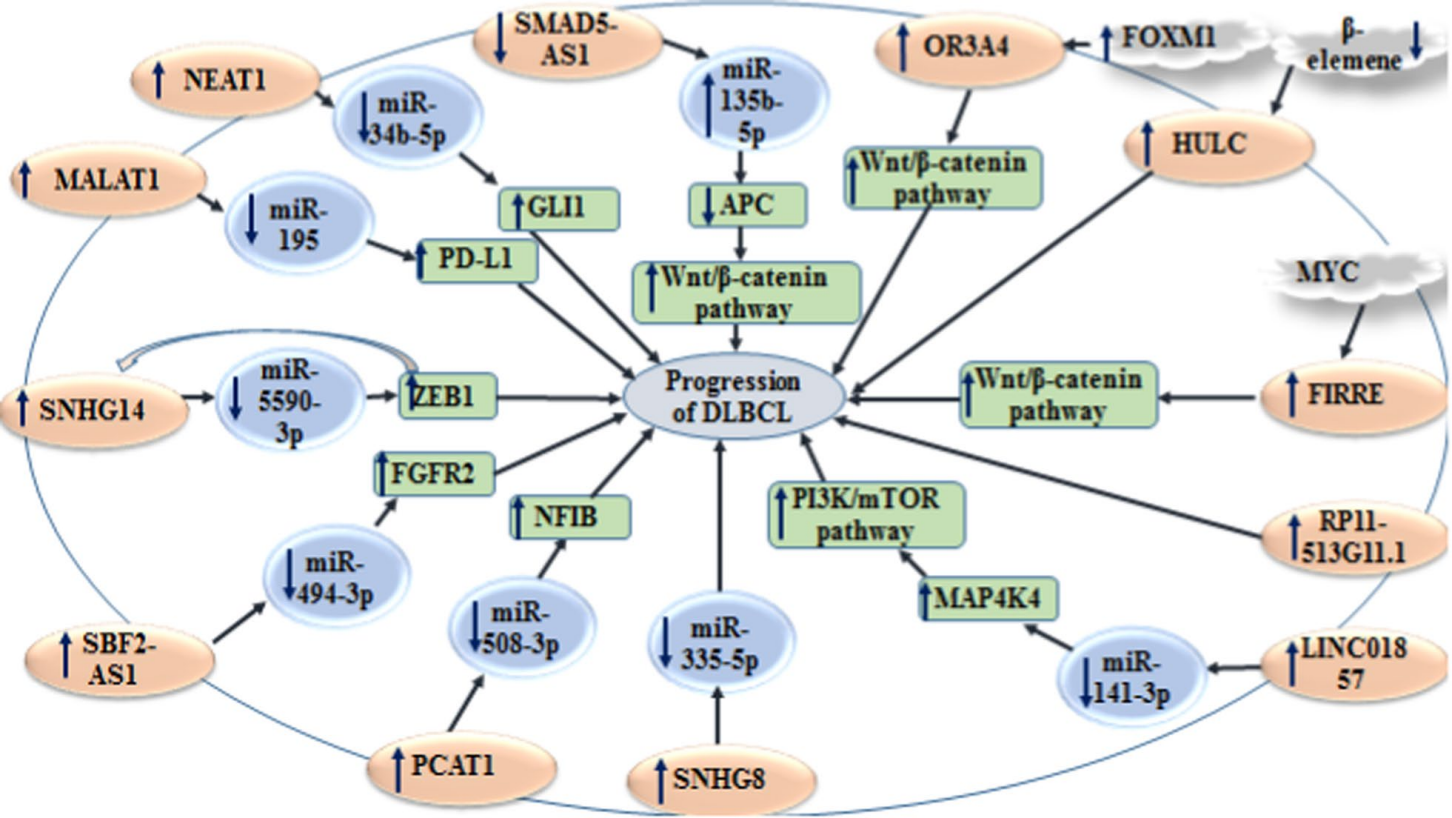
Several lncRNAs can affect availability of miRNAs, thus influencing progression of DLBCL. Detailed information about these lncRNAs is shown in Table 2.

NEAT1 is another lncRNA whose expression has been enhanced in DLBCL tissues and cell lines parallel with up-regulation of GLI1 and down-regulation of miR-34b-5p. NEAT1 silencing or miR-34b-5p up-regulation could inhibit proliferation and enhance apoptosis of these cells. In fact, NEAT1 acts as a competing endogenous RNA (ceRNA) to regulate expression the miR-34b-5p/GLI1 axis. Besides, MYC has been shown to modulate NEAT1 expression through directly binding to promoter of NEAT1 [92]. Figure 2 shows the interactions between lncRNAs and miRNAs in the context of DLBCL.

**Table 2:** LncRNAs and B cell functions

lncRNA	Expression pattern	Disease	Sample	Cell line	Interaction	Signaling pathway	Function	References
*Human studies/mixed studies*								
SNHG14	↑	DLBCL	38 pairs of B cell lymphoma tissues and ANCTs, BALB/c mice	GM12878, 293 T, A20, OCI-LY7, DB, U2932, and FARAGE	↓ miR-5590-3p, ↑ ZEB1, PD-1/PD-L1 checkpoint	–	∆ SNHG14: ↓ proliferation, migration and EMT process There is a positive feedback loop between SNHG14 and ZEB1 to promote DLBCL	[90]
MALAT1	↑	DLBCL	37 patients with DLBCL	OCI-Ly10 human DLBCL cell line, CD8 + T cells	↓ miR-195, ↑ PD-L1 and CD8	Ras/ERK signaling pathway	∆ MALAT1: ↓ proliferation, migration and immune escape ability, EMT-like process, ↑ apoptosis	[91]
CRNDE	↑	BCP-ALL	BM biopsies from 26 patients with BCP-ALL and BM biopsies from 15 patients with unexplained thrombocytosis or anemia as controls	NALM-6, RS4;11 CEMO-1, CCRF-SB, and SUP-B15 BCP-ALL cell lines	↓ miR-345-5p, ↑ CREB	_–	∆ CRNDE: ↓ proliferation, ↑ apoptosis	[93]
AL133346.1	↑	B-ALL	GEO dataset: GSE128254	–	↑ CCN2	–	It was found that either AL133346.1 regulates CCN2 expression in cis; or AL133346.1 and CCN2 are regulated by the same regulatory elements	[94]
NEAT1	↑	DLBCL	30 patients with DLBCL and 30 healthy controls	OCI-Ly1, OCI-Ly8, OCI-Ly10 and SUDHL-4 DLBCL cell lines	↓ miR-34b-5p, ↑ GLI1	–	∆ NEAT1: ↓ proliferation, ↑ apoptosis	[92]
SMAD5-AS1	↓	DLBCL	11 patients with DLBCL and 11 healthy controls, BALB/c-nude mice	TMD8, U2932, GM12878, HEK-293, OCI-Ly3, WSU-FSCCL, JeKo-1, L428, and Raji	↑ miR-135b-5p, ↓ APC	↑ Wnt/β-catenin pathway	↑ SMAD5-AS1: ↓ proliferation, ↑ apoptosis	[97]
OR3A4	↑	DLBCL	58 patients with DLBCL and healthy controls	2932, SU-DHL-6, SU-DHL-4, OCL-LY-7, OCL-LY- 10 DLBCL cell lines and WIL2 human B lymphocyte	↑ FOXM1	↑ Wnt/β-catenin signaling pathway	∆ OR3A4: ↓ proliferation and ↑ apoptosis OR3A4 is upregulated by FOXM1	[98]
FIRRE	↑	DLBCL	70 pairs of DLBCL patient samples and healthy controls	2932, SU-DHL-6, SU-DHL-4, OCL-LY-7, OCL-LY-10 human DLBCL cell lines and WIL2S one normal B-cell line	MYC	↑ Wnt/β-catenin signaling pathway	↑ proliferation and ↓ apoptosis	[99]
RP11-513G11.1	↑	DLBCL	93 patients with DLBCL and 62 healthy controls	–	–	–	Patients with high expression levels of RP11-513G11.1 showed shorter PFS and OS	[100]
lnc-290	↑ in B cells stimulated by LPS	inflammation and tissue damage	female C57Bl/6 mice	GFP + cells	CD69/CD86, LPS/TLR4 signaling pathway	NF-κB/ERK pathways	∆ lnc-290: ↓ growth of B cells, ↓ cell differentiation and ↓ immunoglobulin production, ↓ B cell activation by blocking the LPS/TLR4 signaling pathway	[101]
LINC01857	↑	DLBCL	TCGA and GTEX databases, GEO datasets	HCC1395, CYP6D, OCI-Ly3, and Raji	↓ miR-141-3p, ↑ MAP4K4	PI3K/mTOR pathway	↑ proliferation, ↑ EMT process and ↑ cell cycle progression, ↓ apoptosis	[102]
TEX41	↑ in B-ALL	B-ALL	79 patients with B-ALL, 25 patients with T-cell ALL and 38 acute myeloid leukemia	RS4;11 cells	p53 and p21	–	↑ proliferation, ↑ cell growth and ↑ cell cycle progression	[103]
AFAP1-AS1	↑	GCB-DLBCL	48 patients with DLBCL	OCI-ly1 and OCI-ly19 GCB-DLBCL cell lines	SFPQ, NONO, SRSF2, SRSF6, and KHSRP	BCR and TNF signaling pathways	∆ AFAP1-AS1: ↓ proliferation, ↑ G0/G1 arrest and ↑ apoptosis Patients with higher expression levels of AFAP1-AS1 had poorer DFS and OS	[104]
PTTG3P	↑	IgAN	patients with IgAN and healthy controls	B cells	↓ miR-383, cyclin D1 and ki-67, IL-1β and IL-8	–	↑ PTTG3P: ↑ B cell growth and ↑ cyclin D1 and ki-67 expression, ↑ IL-1β and IL-8 production	[95]
SNHG8	↑	DLBCL	–	GM12878 human B lymphocytes and OCI-Ly10, OCI-Ly7, OCI-Ly3, and U2932 human DLBCL cell lines	↓ miR-335-5p	–	∆ SNHG8: ↓ proliferation, ↓ colony formation and ↑ apoptosis	[105]
PCAT1	↑	DLBCL	48 pairs of DLBCL tissues and ANCTs	OCI-LY-7, OCI-LY-7, TMD8 and U2932 DLBCL cell lines, IM-9 human peripheral blood B-lymphocyte	↓ miR-508-3p, ↑ NFIB	–	↑ PCAT1: ↑ proliferation, ↑ migration and ↑ invasion	[106]
SBF2-AS1	↑	DLBCL	50 patients with DLBCL	OCI-LY-3, OCI-LY-7, OCI-LY-10, SU-DHL-4 and SU-DHL − 6 and HEK293 cells	↓ miR-494-3p, ↑ FGFR2	–	∆ SBF2-AS1: ↓ viability and ↓ growth	[107]
SNHG14	↑	DLBCL	21 patients with DLBCL and 21 healthy controls	GM12878, OCI-LY-7, ABC, OCI-LY-3 and RCK-8	↓ miR-152-3p	–	↑ growth, migration, and EMT-like processes, ↓ apoptosis	[108]
LINC00908	↑	DLBCL	28 pairs of DLBCL tissues and ANCTs, female BALB/c nude mice	GM12878 human lymphoblastoid B cell and OCI-LY7, DB, U2932, and FARAGE human DLBCL cells	miR-671-5p	–	∆ LINC00908: ↓ proliferation and invasion, tumor growth	[109]
TCONS_ 00,022,357-XLOC_010919	↑	GD	Peripheral blood from 34 patients with GD, and 34 healthy controls	CD19 + B cells from 21 healthy individuals and 24 GD patients, PBMCs	TCL1A	–	TCONS_ 00,022,357-XLOC_010919 regulated TCL1A, and TCL1A is involved in B-cell proliferation	[110]
n335641	↑	GD	Peripheral blood from 34 patients with GD, and 34 healthy controls	CD19 + B cells from 21 healthy individuals and 24 GD patients, PBMCs	TCL1A	–	n335641 regulates TCL1A, and TCL1A is involved in B-cell proliferation	
n337845	↓	GD	Peripheral blood from 34 patients with GD, and 34 healthy controls	CD19 + B cells from 21 healthy individuals and 24 GD patients, PBMCs	SH2D1A	–	n337845 regulates SH2D1A, and SH2D1A is involved in B-cell proliferation	
ZEB1-AS1	↑	B-ALL	30 with B-ALL and 30 healthy controls	hBMSC-TERT	–	IL-11/STAT3 pathway	∆ ZEB1-AS1: ↓ proliferation and IL-11 production High expression levels of ZEB1-AS1 showed poor prognosis of B-ALL patients ZEB1-AS1 promoted IL-11 stability	[111]
UCA1	↑	DLBCL	38 patients with DLBCL and 38 healthy controls	GM12878, JeKo-1, TMD8, U2932, OCI-Ly-10 and OCI-Ly-7 cell lines and U2932	↓ miR-331-3p	–	∆ UCA1: ↓ proliferation, viability, migration and invasion	[112]
LAMP5-AS1	↑ in MLL leukemia patients than that in the MLL-wt leukemia	MLL leukemia	58 patients with MLL leukemia and 163 MLL-wt leukemia, NOD-SCID mice	MOLM13, THP1, MV4-11, RS4-11, and HEK293T human MLL leukemia cells	DOT1L	–	∆ LAMP5-AS1: ↓ colony formation and ↑ differentiation of primary MLL leukemia CD34 + cells Patients with high levels of LAMP5-AS1 showed a reduced 5-year leukemia-free survival LAMP5-AS1 increased the methyltransferase activity of DOT1L	[113]
LINC00152	↑	Gastric cancer	30 pairs of GC tissues and ANCTs, male BALB/c nude mice	RGM‐1 human epithelial cells of gastric mucosa, and human BGC‐823 GC cell	Bcl-2	–	↑ migration and invasion, ↓ apoptosis	[114]
HCP5	↑	DLBCL	48 patients with DLBCL and 14 RLH samples	OCI-LY7 and OCI-LY3 human DLBCL cell lines	↓ miR-27b-3p, ↑ MET	–	∆ HCP5: ↓ proliferation, ↑ apoptosis Geniposide treatment: ↓ HCP5	[115]
PEG10	↑	DLBCL	25 patients with DLBCL and 25 healthy controls	SU-DHL-8 and OCI-LY-8 DLBCL cell lines	↓ miR-101-3p, ↑ KIF2A	–	∆ HCP5: ↓ proliferation, migration and invasion, ↑ apoptosis	[116]
GAS5	↓	DLBCL	–	OCI-Ly3 and TMD8 cells	↑ miR-18a-5p, ↓ RUNX1	–	∆ GAS5: ↓ proliferation, ↓ G1 arrest, ↑ apoptosis	[117]
TUG1	↑	DLBCL	15 tumor tissues and venous blood from DLBCL patients, 15 patients with RLH as controls, female BALB/c athymic nude mice	OCI-LY7, and OCI-LY3 human DLBCL cell lines, IM-9I normal B lymphocyte	↑ MET	–	∆ TUG1: ↓ proliferation and tumor growth	[118]
SNHG12	↑	DLBCL	80 patients with activated B-cell like DLBCL, 80 patients with RLH as controls, male BALB/c nude mice	OCI-LY7, and OCI-LY3 human DLBCL cell lines, IM-9I normal B lymphocyte	↓ miR-195	–	∆ SNHG12: ↓ cell growth, ↓ migration, and ↓ invasion	[119]
PANDA	↓	DLBCL	114 patients with DLBCL and 114 healthy controls	U2932, SUDHL-6, SUDHL-3, OCI-Ly3, and OCI-Ly8 human DLBCL cell lines and WIL2S normal B-cell line	p53	MAPK/ERK signaling pathway	↑ G0/G1 cell cycle arrest and ↓ proliferation through silencing MAPK/ERK signaling pathway Low levels of PANDA were associated with poorer clinical outcome and lower OS in DLBCL patients	[120]
GAS5	↓	B lymphocytic leukemia	30 patients with human B lymphocytic leukemia, 30 healthy controls	RAMOS, ST486, Raji, and Farage human B lymphocytic leukemia cell lines and IM9 normal B lymphocytic cell line	miR-222	–	↑ GAS5: ↓ proliferation, and ↓ invasion, ↑ apoptosis and ↑ G1 phase arrest	[121]
DBH-AS1	↑	DLBCL	26 patients with DLBCL	RCK‐8, OCI‐LY‐3, OCI‐LLY‐7, and OCI‐LY‐ 10 human DLBCL cell lines and IM‐9 human peripheral blood B‐lymphocyte	BUD13, FN1	–	∆ DBH-AS1: ↓ proliferation, ↓ invasion, and ↓ migration DBH-AS1 regulated FN1 expression by recruiting BUD13	[122]
ROR1-AS1	↑	MCL	5 patients with MCL and 5 healthy controls	Mino, Granta, JVM2 and Z138 MCL cell lines, HEK-293 T cell line	↓ P16, and SOX11 EZH2 and SUZ12 of polycomb repressive complex-2	–	↑ ROR1-AS1: ↑ cell growth and ↓ sensitivity to the treatment with drugs ibrutinib and dexamethasone ROR1-AS1 is involved in epigenetic regulation of gene transcription through EZH2/PRC2 complex	[123]
LHFPL3-AS1	↑	Melanoma	461 tumor tissues and 558 normal tissues, BALB/c nude mice	Melanoma stem cells and non-stem cells from MDA-MB-435 cells	↑ PTBP1, ↓ miR-181a-5p, ↑ Bcl-2	–	∆ LHFPL3-AS1: ↓ proliferation, ↑ apoptosis of melanoma stem cells	[124]
NONHSAG026900	↓	DLBCL	GEO dataset GSE12453 including 11 patients with DLBCL and 25 healthy controls and GSE56315, GSE11318, GSE23501, GSE53786, GSE10846, and GSE31312	–	–	–	↓ proliferation and cell cycle progression	[125]
SNHG16	↑	DLBCL	DLBCL tissues (21 GCB and 27 non‐GCB) and 14 RLH tissues as controls, male NOD/SCID mice	OCI‐LY7 and OCI‐LY3	↓ miR-497-5p, ↑ PIM1	–	∆ SNHG16: ↓ proliferation, growth, and cell cycle progression, ↑ apoptosis	[126]
NEAT1–1	↑ in DLBCL tissues	DLBCL	64 patients with DLBCL and 15 patients with lymphnoditis	OCI-Ly1 and SUDHL-4 DLBCL cell lines	–	–	∆ NEAT1_1: ↓ viability and migration, ↑ apoptosis High levels of NEAT1_1 were correlated with stage, IPI, extranodal site involvement and drug response	[127]
TUC338	↑	DLBCL	102 pairs of DLBCL and normal tissues, serum specimens of 35 patients with DLBCL and 35 healthy controls, BALB/c nude mice	U2932 and OCI-Ly3 DLBCL cell lines	↓ miR-28-5p, ↑ EGFR	↑ PI3K/AKT signaling,	∆ TUC338: ↓ proliferation and chemotherapy resistance to Adriamycin, ↑ apoptosis Patients with high TUC338 showed shorter survival time	[128]
LINC00857	↑	DLBCL	87 pairs of DLBCL tissues and ANCTs	HMy2.CIR lymphoblast cell line, SU-DHL-6, SU-DHL-4 and SU-DHL-10 DLBCL cell lines	↓ miR-370-3p, ↑ CBX3	–	↑ LINC00857: ↑ proliferation and cycle progression, ↓ apoptosis ∆ TUC338: ↓ proliferation and, ↑ apoptosis	[129]
Lnc-IRF2-3 and Lnc-ZNF667-AS1	↑	B-CLL	135 patients with B-CLL and 30 healthy controls	–	–	–	Patients with high levels of Lnc-IRF2-3 had a significant decrease in OS and PFS High levels of Lnc-IRF2-3 and Lnc-ZNF667-AS1 were associated with poor survival	[130]
LINC00963	↓	DLBCL	GTEx and TCGA databases (normal N = 337, tumor T = 48), nude mice	SUDHL4, OCI-Ly1, HBL1 and OCI-Ly3 DLBCL cell lines and GM12878 Non-cancerous human B lymphocytes	↑ miR-320a, ↓ XBP1	–	↑ LINC00963: ↓ proliferation, and tumor growth, ↑ apoptosis and autophagy	[131]
LEF1-AS1	↑	CLL	–	primary CLL cells and normal B cells	↑ LEF1	–	↑ LEF1-AS1: ↑ proliferation and ↓ apoptosis	[132]
PVT1	↑	MM	137 patients with MM and 62 patients with MGUS, and 21 control patients with lymphoma	KMS11, KMS12PE, KMS12BM, KMS26, KMM1, OPM2, RPMI8226	↑ MYC, BRD4	–	High levels of PVT1 were positively correlated with disease progression JQ1 (BRD4 inhibitor): ↓ proliferation and ↓ expression levels of MYC and PVT1	[133]
BALR-2	↑	B-ALL	160 patients with B-ALL	RS4;11 and MV4;11, Reh, 697, Nalm-6, and 70Z/3 murine pre-B-cell leukemic cell line, and HEK 293 T cell line	–	Glucocorticoid response pathway	∆ BALR-2: ↓ proliferation, ↑ apoptosis and sensitivity to prednisolone treatment prednisolone treatment: ↓ BALR-2 expression	[134]
FAS-AS1	↓	Lymphoma	–	Granta-519 cells and Peripheral blood B-lymphocytes from healthy donors’ blood	↑ sFas, RBM5, ↑ EZH2,	–	FAS-AS1 could regulate alternative splicing of Fas in lymphomas Expression of FAS-AS1 could repress by EZH2	[135]
LUNAR1	↑	DLBCL	87 patients with DLBCL and 28 samples with reactive lymph nodes as controls	OCI-LY-3, OCI-LY-7, OCI-LY-10, SU-DHL-4, SU-DHL-6 and RCK-8 DLBCL cell lines	–	–	∆ LUNAR1: ↓ proliferation LUNAR1 expression was found to serve as an independent predictor for OS and PFS	[136]
HOTAIR	↑	DLBCL	50 lymph node samples from patients with DLBCL and 20 samples with reactive lymph nodes as controls	RCK-8, OCL-LY-10, OCL-LY-7, SU-DHL-6 and SU-DHL-4 DLBCL cell lines	–	PI3K/AKT/NF-κB signaling pathway	∆ HOTAIR: ↓ growth, cell cycle progression, ↑ apoptosis	[137]
RP11-530C5.1	↑	MS	GEO database and GSE21942, 50 MS patients and 25 controls	–	PAWR	–	–	[96]
AL928742.12	↓	MS	GEO database and GSE21942, 50 patients with MS and 25 controls	–	IGHA2	–	–	
PEG10	↑	DLBCL	107 patients with DLBCL and 46 samples with reactive lymph nodes as controls	OCI-LY-3, OCI-LY-7, OCI-LY-10, RCK-8, SU-DHL-4 and SU-DHL-6 DLBCL cell lines	–	–	∆ PEG10: ↓ growth, ↑ apoptosis PEG10 levels were significantly associated with B symptoms, IPI score, CHOP-like treatment and rituximab	[138]
HULC	↑	DLBCL	142 patients with DLBCL and 60 samples with reactive lymph nodes as controls	OCI–LY–3, OCI–LY–7, OCI–LY–10, SU–DHL–4, SU–DHL–6 and RCK–8 human DLBCL cell lines	–	–	∆ HULC: ↓ proliferation, ↑ apoptosis HULC was strongly associated with Ann Arbor stages, B symptoms, CHOP-like treatment, rituximab and IPI	[139]
lincRNA-p21	↓	DLBCL	105 patients with DLBCL	SU-DHL-2, OCI-LY-3, OCI- LY-10, SU-DHL-4 and OCI-LY-7 human DLBCL cell lines	–	–	↑ lincRNA-p21: ↓ proliferation and cycle progression Patients with high expression levels of lincRNA-p21 showed a favorable OS and PFS	[140]
*Murine studies*								
BALR-6	↑	B-ALL	Post bone marrow transplant, blood, bone marrow, thymus and spleen were collected from the mice	RS4;11 and MV, Reh, 697, Nalm-6, 70Z/3 murine pre B-cell leukemic cell line, and the HEK 293 T cell line	SP1, CREB1	–	∆ BALR-6: ↓ proliferation, ↑ apoptosis ↑ BALR-6: ↑ survival, proliferation and expansion of hematopoietic progenitor populations in vivo	[141]
RP11-301G19.1	↑	MM	Female BALB/c-nude mice	U266, RPMI8226, OPM-2, MM-1S, NCI- H929 MM cell lines and 293 T normal plasma cells	↓ miR-582-5p, ↑ HMGB2	PI3K/AKT signaling pathway	∆ RP11-301G19.1: ↓ proliferation and cell cycle progression, ↑ apoptosis	[142]
HULC	↑	DLBCL	Male BALB/C mice	SU-DHL-8, SU-DHL-10 human DLBCL cell lines	β-elemene	–	↑ HULC: ↓ apoptosis	[143]
lnc00492	↑	–	lnc00492−/− and lnc00492 + / + mice	B220 + B cells, MZ B-cells	↓ CTBP1	Notch2 signaling pathway	Lnc00492 is necessary for marginal zone B-cell development	[144]
MALAT-1	↑	DLBCL	Female BALB/c-nu/nu nude mice	IM-9 cells, B lymphocytes IM-9I from healthy people and Farage, Pfeiffer, Raji, Daud, Ly1, Ly3, Ly8, and Ly10 from patients with DLBCL	↓ LC3-II/LC3-I, ↑ p62	–	∆ U MALAT-1: ↓ migration, survival rate, the proportion of cells in S and G2/M phase, and tumor volume and weight, ↑ the proportion of cells in G0/G1 phase	[145]
NEAT1	↑	SLE	Lupus-prone MRL/lpr mice	PBMCs, B220 + B cells, G-MDSCs or M-MDSCs from MRL/lpr mice	BAFF	IFN-I signaling	↑ promotion of G-MDSCs ∆ NEAT1: ↓ lupus symptoms and inhibits IFN-I signaling activation	[146]

CRNDE has been shown to be up-regulated in the bone marrow of B-cell precursor acute lymphoblastic leukemia patients and related cell lines. CRNDE silencing has decreased cell proliferation and enhanced cell apoptosis in these cells. Functionally, CRNDE could bind with to miR-345-5p and down-regulate its expression, thus affecting expression of CREB. Notably, in vivo studies have shown that CRNDE silencing increases survival of mice models of this type of leukemia [93].

In addition to this type of studies, expression patterns of lncRNAs have been compared between cancer cells and non-cancerous controls using high throughput methods. For instance, Cuadros et al. have reported differential expression of 48 lncRNAs between pediatric B-ALL and normal bone marrow specimens. They have recognized AL133346.1/CCN2 as the most relevant lncRNA/mRNA pair in this type of malignancy. Expression of AL133346.1/CCN2 pair has been enhanced in B-ALL specimens [94].

Expression of PTTG3P has been shown to be up-regulated in samples obtained from patients with IgA nephropathy compared with normal samples. Notably, expression of PTTG3P in urine samples has been correlated with expression of PTTG3P in intra-renal samples of IgA nephropathy cases. Up-regulation of PTTG3P has stimulated B cell growth and increased expressions of cyclin D1 and ki-67. In addition, its up-regulation of PTTG3P has led to induction of IL-1β and IL-8 release. PTTG3P up-regulation could suppress expression of miR-383 in B cells. Taken together, PTTG3P could increase B cell growth and IL-1β and IL-8 release through influencing expression of miR-383. Through this effect, PTTG3P contributes in the pathogenesis of IgA nephropathy [95].

Expression of lncRNA RP11-530C5.1 has been shown to be higher in relapsing MS patients, compared to remitting MS patients and healthy subjects, whereas expression of AL928742.12 has been decreased. Notably, expression levels of RP11-530C5.1 and AL928742.12 have been correlated with PAWR and IGHA2 levels, respectively [96].

Table 2 shows the impact of lncRNAs in B cell functions.

## Contribution of circRNAs in the regulation of B cell-related disorders

The impact of circRNAs on B cell functions has been mostly assessed in the context of DLBCL. For instance, circ_OTUD7A expression has been found to be increased in DLBCL. Its silencing has suppressed proliferation and metastasis of DLBCL, induce cell cycle arrest and enhance their apoptosis. Mechanistically, circ_OTUD7A acts as a sponge for miR-431-5p and miR-431-5p to further regulate expression of FOXP1 [147].

Another study has shown that up-regulation of circCFL1 in DLBCL cells leads to reduction of miR-107 levels and subsequent up-regulation of HMGB1 in these cells. Functional studies have revealed that circCFL1 could directly bind with miR-107 and release HMGB1 from inhibitory effects of this miRNA. Up-regulation of circCFL1 increases migration and proliferation of DLBCL cells [148].

Circ-APC is another circRNA which is produced from APC and suppress proliferation of DLBCL cells through decreasing activity of Wnt/β-catenin pathway. This effect is exerted through its interaction with TET1 and miR-888 [149].

The impact of circRNAs has also been investigated on progression of leukemia. For instance, circ_0132266 has been shown to be down-regulated in chronic lymphocytic leukemia. This down-regulation has lead to enhancement of viability of these cells via influencing activity of miR-337-3p/PML axis [150]. Table 3 shows the effects of circRNAs in the pathogenesis of B cell-related disorders.

**Table 3:** CircRNAs and B cells functions

circRNA	Expression pattern	Disease	Sample	Cell line	Interaction	Signaling pathway	Function	References
*Human studies*								
Circ_OTUD7A	↑	DLBCL	50 pairs of DLBCL tissues and ANCTs	U2932, TMD8 and OCI-Ly3 LBCL cell lines and GM12878 normal human B lymphocytes	↓ miR-431-5p, ↑ FOXP1	–	∆ Circ_OTUD7A: ↓ proliferation, metastasis, ↑ cell cycle arrest and apoptosis	[147]
circ-APC	↓	DLBCL	80 pairs of DLBCL and para-cancerous tissues, plasma samples from 27 DLBCL patients and 16 healthy controls, nude mice	SUDHL-3, U2932, TMD8, OCI-Ly3 and L428 human DLBCL cell lines and GM12878 normal human B lymphocytes	miR-888, APC, DNA demethylase TET1	Wnt/β-catenin signaling pathway	↑ CircCFL1: ↓ proliferation and tumor growth	[149]
circBCL11B	↑	AML	61 patients with AML and 16 healthy samples, GEO dataset: GSE137851	–	–	–	∆ circBCL11B: ↓ proliferation, ↑ apoptosis	[151]
circCDYL	↑	MCL	18 patients with MCL and 17 healthy controls	HEK293T cells and Z138 human MCL cell line	five miRNAs (hsa-miR-129-5p, hsa-miR-3163, hsa-miR-4662a-5p, hsa-miR-101-3p, and hsa-miR-186-5p), three lncRNAs (MALAT1, NEAT1, and XIST), and five mRNAs (NOTCH1, FMR1, ABCB1, TWIST1, and VEGFA)	–	∆ circCDYL: ↓ proliferation	[152]
circ_0132266	↓	CLL	30 patients with CLL and 30 healthy controls	MEC-1, JVM-3 and HEK-293 T	↑ miR-337-3p, ↓ PML	–	↑ circ_0132266: ↓ proliferation	[150]
circ_0005774	↑	AML	20 patients with AML and 20 healthy controls	HL-60 and NB4 cells	↓ miR-192-5p, ↑ ULK1	–	∆ circ_0005774: ↓ proliferation and viability, ↑ apoptosis	[153]
circ-Smad5	↓	DLBCL	–	JB6 and 293 T cell lines	–	Wnt/β-catenin/Lef1 signaling pathway	∆ circ-Smad5: ↑ cell cycle progression and activated Wnt/β-catenin/Lef1 signaling pathway	[154]
circ_0009910	↑	AML	35 patients with AML and 35 healthy controls	HL-60 and MOLM-13	↓ miR-5195-3p and ↑ GRB10	–	∆ circ_0009910: ↓ proliferation and cell cycle progression, ↑ apoptosis	[155]
circ-CBFB	↑	CLL	47 patients with CLL and 21 healthy controls	HEK293T and MEC-1 human CLL cell line	↓ miR-607, ↑ FZD3	↑ Wnt/β-catenin pathway	∆ circ-CBFB: ↓ proliferation and cell cycle progression, ↑ apoptosis	[156]
*Murine studies*								
CircCFL1	↑	DLBCL	female BALB/c nude mice	OCI-Ly7 and OCI-Ly3 human DLBCL cell lines	↓ miR-107, ↑HMGB1	–	↑ CircCFL1, ↑ proliferation, migration, tumor volume and weight	[148]

ANCTs, adjacent non-cancerous tissues; AML, acute myeloid leukemia; HSPC, hematopoietic stem and progenitor cell; MCL, Mantle cell lymphoma; CLL, chronic lymphocytic leukemia

## Discussion

Accumulating evidence suggest the role of non-coding RNAs in the development of normal B cells as well as lymphomagenesis. Since they are have a highly cell type specific signature, these transcripts have been suggested as potential biomarkers for diverse clinical situations [157].

LncRNAs particularly those related with p53 or MYC pathways have also applications as therapeutic targets [157]. These transcripts could act as sponges for miRNAs, thus influencing expressions of their target genes. SNHG14/miR-5590-3p, MALAT1/miR-195, CRNDE/miR-345-5p, NEAT1/miR-34b-5p, SMAD5-AS1/miR-135b-5p, PTTG3P/miR-383, SNHG8/ miR-335-5p, PCAT1/miR-508-3p, SBF2-AS1/miR-494-3p, SNHG14/ miR-152-3p and LINC00908/miR-671-5p are among lncRNA/miRNA axes which are involved in the regulation of B cells. Ras/ERK, Wnt/β-catenin pathway, NF-κB/ERK, PI3K/mTOR, BCR, TNF, IL-11/STAT3, IFN-I, Notch2, MAPK/ERK, PI3K/AKT and glucocorticoid response pathways are among pathways that are regulated by lncRNAs in this context.

CircRNAs that regulate function of B cells are mostly associated with Wnt/β-catenin signaling pathway. They can also serve as sponges for miRNA. For instance, circ_OTUD7A/miR-431-5p, circCFL1/miR-107, circ-APC/miR-888, circ_0132266/miR-337-3p, circ_0005774/miR-192-5p, circ_0009910/miR-5195-3p and circ-CBFB/miR-607 are among important circRNA/miRNA axes in regulation of proliferation of B cells.

Finally, miRNAs that are involved in the pathogenesis of B cell-related disorders can modulate NF-κB, TGF-β, BCR, TAK1/IKKα-IKKβ/IκBα and MAPK/p65 signaling pathways.

Cumulatively, different classes of non-coding RNAs interact with each other to modulate function of B cells. Notably, non-coding RNAs have also interactions with immune check point proteins in the context of B cell disorders.

## Conclusion

The observed interaction between non-coding RNAs and immune check point proteins suggests the importance of these transcripts as targets for immunotherapeutic approaches. Moreover, several lncRNAs, circRNAs and miRNAs have been found to affect proliferation of B cells, thus being involved in the pathogenesis of B cell-related disorders, particularly malignant disorders. The observed correlations between expression levels of these transcripts and clinic-pathological parameters further emphasize their role in the carcinogenic processes.

Understanding the impact of non-coding RNAs in B cell-related malignancies would provide new avenues for targeted therapies.

## Data Availability

The analyzed data sets generated during the study are available from the corresponding author on reasonable request.
